# Multiple Sclerosis Affects Skeletal Muscle Characteristics

**DOI:** 10.1371/journal.pone.0108158

**Published:** 2014-09-29

**Authors:** Inez Wens, Ulrik Dalgas, Frank Vandenabeele, Maartje Krekels, Lotte Grevendonk, Bert O. Eijnde

**Affiliations:** 1 REVAL - Rehabilitation Research Center, BIOMED - Biomedical Research Institute, Faculty of Medicine and Life Sciences, Hasselt University, Hasselt, Belgium; 2 Section of Sport Science, Dep. Public Health, Aarhus University, Aarhus C, Denmark; University of Minnesota Medical School, United States of America

## Abstract

**Background:**

The impact of multiple sclerosis (MS) on skeletal muscle characteristics, such as muscle fiber cross sectional area (CSA), fiber type proportion, muscle strength and whole muscle mass, remains conflicting.

**Methods:**

In this cross sectional study, body composition and muscle strength of the quadriceps were assessed in 34 MS (EDSS: 2.5±0.19) patients and 18 matched healthy controls (HC). Hereafter a muscle biopsy (m.vastus lateralis) was taken.

**Results:**

Compared to HC, mean muscle fiber CSA of all fibers, as well as CSA of type I, II and IIa fibers were smaller and muscle strength of the quadriceps was lower in MS patients. Whole body composition was comparable between groups. However, compared to HC, the biopsied leg tended to have a higher fat percentage (p = 0.1) and a lower lean mass (p = 0.06) in MS patients.

**Conclusion:**

MS seems to negatively influence skeletal muscle fiber CSA, muscle strength and muscle mass of the lower limbs of mildly affected MS patients. This emphasises the need for rehabilitation programs focusing on muscle preservation of the lower limb.

**Trial Registration:**

ClinicalTrials.gov NCT01845896

## Introduction

Multiple sclerosis (MS) is characterised by complex and heterogeneous symptoms, often leading to reduced quality of life [1] and impaired functional capacity [2]. The latter is related to reduced muscle strength of predominately the lower limbs [3], [4]. The mechanisms underlying the observed strength deficits are of muscular [5]–[7] as well as neural origin [8], [9].

At the whole muscle level, a number of studies have examined skeletal muscle characteristics of MS patients, with some studies [5], [6], [10], but not all [7], [9], [11], reporting loss of muscle mass and decreased [6]–[9], [12], [13] or comparable [5] maximal muscle strength. Neurologically, central impairments of the central motor function are reported [9]. At present, it remains unknown whether the reported observations are consequences of the disease *per se*, are caused by inactivity or are affected by a combination of both.

At the cellular level the impact of MS on muscle fiber cross sectional area (CSA) and muscle fiber proportion remains conflicting. On the one hand two small studies (n = 9 [5] and n = 6 [6]) have reported reduced muscle fiber size in MS patients compared to healthy controls (HC) [5], [6], while one study indicated alterations, but did not include a direct comparison to HC [14]. On the other hand, one small study (n = 7 [7]) did not report any alterations [7], clearly suggesting a need for well-powered studies to clarify the muscular influence of MS. Furthermore, corresponding to immobilized healthy controls [15]–[17], a shift from type I to type IIa and IIax fibers was reported in a small group of MS patients by Kent-Braun et al. [5], but this was not confirmed by others [6], [7], [9].

To clarify the heterogeneous results of the existing literature in small groups of MS patients, the present cross sectional study aimed to investigate the effect of MS on muscle fiber CSA and proportion, muscle strength and body composition in a larger group of MS patients, compared with HC. It was hypothesized that MS would negatively affect skeletal muscle characteristics.

## Methods

### Subjects

Thirty-four MS patients diagnosed according to the McDonald criteria (EDSS range 0, 5–6) and 18 matched healthy controls (HC), aged >18 years, were included following written informed consent, providing a ∼2∶1 match for gender, age and body mass index (BMI). Subjects were excluded if they had other chronic disorders (cancer, cardiovascular, pulmonary and/or renal diseases), were pregnant, participated in another study and, in case of MS patients, have had an acute MS exacerbation 6 months prior to the start of the study. The study was approved by the ethical committee of Hasselt University and Jessa Hospital Hasselt and was part of a study registered at ClinicalTrials.gov (NCT01845896). All tests were performed in accordance with the Declaration of Helsinki.

### Primary outcome measure

#### 1. Skeletal muscle fiber cross sectional area and fiber type proportion

Muscle biopsies were obtained from MS patients and HC from the middle part of the m.vastus lateralis (Bergström needle technique), by an experienced medical doctor. Because we aimed to evaluate the impact of MS on muscle fiber characteristics, the muscle biopsies of the MS patients were obtained from the weakest leg, as assessed by a preceding isometric muscle strength test performed on an isokinetic dynamometer (System 3, Biodex, ENRAF-NONIUS, New York, USA). The biopsied leg of the HC was randomized, since muscle strength associated with each leg (left vs. right or dominant vs. non-dominant) is equal in healthy persons [18]–[22]. The collected tissue was freed from connective tissue and immediately embedded in Tissue-Tek, frozen in isopentane cooled with liquid nitrogen and stored at -80°C, until further analysis was performed.

Serial transverse sections (9 µm) from the obtained muscle samples were cut at −20°C and stained by means of ATPase histochemistry, after preincubation at pH 4.4, 4.6 and 10.3, essentially following the procedure of Brooke and Kaiser [23]. The serial sections were visualized and analysed using a Leica DM2000 microscope (Leica, Stockholm, Sweden) and a Leica Hi-resolution Color DFC camera (Leica, Stockholm, Sweden) combined with image-analysis software (Leica Qwin ver. 3, Leica, Stockholm, Sweden). This software was able to automatically draw a fiber mask at the stained sections. Afterwards, this mask was fitted manually to the cell borders of the selected fibers. Only fibers cut perpendicularly to their longitudinal axis were used for the determination of fiber size. On average 170±10 fibers were calculated and included in the CSA and fiber type analyses.

Calculation of the fiber CSA was performed for the major fiber types (I, II, IIa and IIx) and for the mean fiber CSA, since the number of fibers expressing the minor fiber types (IIax and IIc) was too small for statistical comparison and CSA calculation.

### Secondary outcome measures

Approximately 1 to 2 weeks before the muscle biopsy was performed, body composition and isometric muscle strength of the quadriceps were assessed from all subjects.

#### 1. Body composition

A Dual Energy X-ray Absorptiometry scan (GE Hologic Series Delphi-A, Vilvoorde, Belgium) was performed. Fat and lean tissue mass were obtained for the whole body as well as for different regions covering the legs, the trunk, the gynoid and the android region. Waist-to-hip fat mass ratio (android fat (g)/gynoid fat (g) ratio) and fat mass of the trunk/fat mass of the limbs ratio were calculated.

#### 2. Isometric muscle strength of the quadriceps

Following 5 min of warming-up on a cycle ergometer and after habituation, the maximal voluntary isometric muscle strength of the knee extensors (45° and 90° knee angle) were measured, as reported elsewhere [24], using an isokinetic dynamometer (System 3, Biodex, ENRAF-NONIUS, New York, USA) in all MS patients and 50% of HC. Briefly, two maximal isometric extensions (4 s), separated by a 30 s rest interval, were performed. The highest isometric extension peak torque (Nm) was selected as the maximal isometric strength. Because we aimed to evaluate the impact of MS on muscle strength, the muscle strength of the weakest, biopsied, leg of MS patients was reported, whereas the tested leg of HC was randomized. Muscle strength of the quadriceps was reported as the mean torque of the knee extensions at 45° and 90°.

### Statistical analysis

All data were analysed using SAS 9.2 software (SAS Institute Inc, Cary, USA). First normality was checked using the Shapiro-Wilk test for all variables. Differences between MS patients and HC were analysed using unpaired t-tests. Correlations were analysed by means of Pearson's correlation analysis. All data are presented as mean ±SE and p<0.05 represents the threshold for statistical significance.

## Results

### Subject characteristics

No differences in general subject characteristics were found between MS patients and HC (Table 1).

**Table 1:** Subject and disease characteristics and overview of secondary outcome measures in MS patients and healthy controls.

	Healthy controls	MS patients	p-value
**Number (f/m)**	18 (13/5)	34 (22/12)	NS
**Age (y)**	47.5±1.9	45.7±1.7	NS
**EDSS**	/	2.5±0.19	/
**Type MS (RR/CP)**	/	26/8	/
**Height (m)**	1.71±0.02	1.70±0.01	NS
**Total body:**			
**Total body weight (kg)**	73.8±3.2	74.4±2.2	NS
**BMI (kg/m^2^)**	25.0±1.0	25.8±0.7	NS
**Total fat mass (kg)**	26.1±2.5	26.4±1.4	NS
**Total fat percentage (%)**	34.5±2.2	36.1±1.4	NS
**Total lean tissue (kg)**	47.9±1.9	46.2±1.5	NS
**Leg muscle biopsy:**			
**Leg mass (kg)**	12.9±0.6	12.3±0.4	NS
**Leg fat mass (kg)**	4.7±0.5	4.9±0.3	NS
**Leg fat percentage (%)**	35.6±2.6	38.9±1.7	0.1
**Leg lean tissue (kg)**	8.2±0.4	7.4±0.9	0.06
**Isometric muscle strength (Nm)**	138±8	108±8	0.04

Data are reported as mean ±SE.

Abbreviations used: MS, multiple sclerosis; m, male; f, female; BMI, body mass index; RR, relapsing remitting; CP, chronic progressive; EDSS, expanded disability status scale; NS, not significant.

### Primary outcome measure

#### 1. Skeletal muscle fiber cross sectional area and fiber type proportion

**Figure 1:**
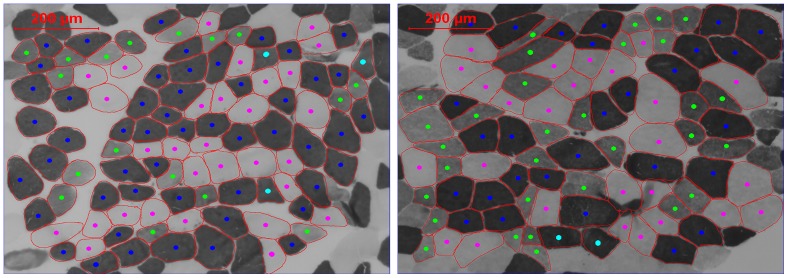
Representative image of fiber type analysis of MS patients (left) and healthy controls (right). Different fiber types are distinguished by color (dark blue: type I, pink: type IIa, green: type IIx, light blue: type IIc). Calculation of the fiber CSA was performed for the major fiber types (I, IIa and IIx) and for the mean fiber CSA, since the number of fibers expressing the minor fiber types (IIax and IIc) was too small for statistical comparison and CSA calculation.

Figure 1 shows a representative image of muscle fiber types of MS patients and HC. Compared to HC, mean muscle fiber CSA, as well as CSA of type I, II, and IIa fibers were significantly smaller in MS patients (p<0.05), whereas muscle fiber CSA of type IIx was comparable between both groups. Furthermore, type II fibers experienced a larger atrophy, compared to type I fibers in MS (p<0.05). Compared to women, men had a higher CSA for almost all fiber types in both HC and MS. Compared to HC, fiber type I proportion tended to be lower in MS (p = 0.1), whereas type IIa proportion tended to be higher (p = 0.1, Table 2).

**Table 2:** Muscle fiber distribution and cross sectional area (CSA) of MS patients and matched healthy controls (HC).

	Healthy controls			MS patients		
	All	Women	Men	All	Women	Men
**Fiber type distribution (%)**						
**Type I**	46.1±2.8	44.7±2.1	49.7±9.2	41.6±2.3 ^b^	45.7±2.6	35.2±3.3 a ^,^ c
**Type IIa**	32.6±2.7	29.0±2.2	41.9±6.5 e	36.4±2.1 ^b^	34.3±2.6 ^b^	39.7±3.3
**Type IIx**	23.2±2.9	27.7±2.9	10.1±3.9 e	21.8±1.9	20.1±2.6 a	24.4±2.5 a
**Fiber CSA (µm^2^)**						
**Mean**	4621±302	4422±321	5660±474 e	3827±200 a	3512±199 a	4326±354 a ^,^ c
*(atrophy compared to HC)*				*(−17.2±4.4%)*	*(−16.8±4.8%)*	*(−23.5±6.5%)*
**Type I**	4880±313	4682±383	5398±514	4109±223 a	3912±226 a	4422±425 ^b,d^
*(atrophy compared to HC)*				*(−15.8±4.6%)*	*(−16.4±4.9%)*	*(−18.1±8.2%)*
**Type II**	4353±332	3875±315	5595±616 e	3502±219 a	3065±193 a	4192±395 a ^,^ c
*(atrophy compared to HC)*				*(−19.5±5.1%)*	*(−20.8±5.1%)*	*(−25.1±7.3%)*
**Type IIa**	4985±342	4448±313	6380±592 e	3862±234 a	3340±193 a	4689±415 a ^,^ c
*(atrophy compared to HC)*				*(−22.5±4.7%)*	*(−24.9±4.4%)*	*(−26.5±6.7%)*
**Type IIx**	3566±277	3360±327	4181±459 ^f^	3165±226	2765±221 ^b^	3766±391 c
*(atrophy compared to HC)*				*(−11.2±6.4%)*	*(−17.7±6.7%)*	*(−9.9±9.7%)*

Data are reported as mean ±SE.

a p<0.05, ^b^p≤0.1, compared to corresponding healthy controls.

c p<0.05, ^d^p≤0.1, comparison between male and female MS patients.

e p<0.05, ^f^p≤0.1, comparison between male and female healthy controls.

### Secondary outcome measures

#### 1. Body composition

Total body composition did not differ between MS patients and HC. In particular, there were no differences between total body weight, adipose and lean tissue mass of MS and HC (Table 1). However, compared to HC, the lower limb, of which the muscle tissue was collected, tended to have a higher fat percentage (p = 0.1) and a lower lean mass (p = 0.06) in the MS patients.

#### 2. Isometric muscle strength of the quadriceps

Compared to HC, MS patients showed reduced isometric muscle strength of the quadriceps of the biopsied leg (−22%, p<0.05, Table 1).

### Correlations

Mean CSA as well as type I, II, IIa and IIx CSA were highly correlated with muscle strength of the quadriceps (r values between 0.70 and 0.81, p<0.05). All muscle fiber types CSA and mean CSA of MS patients and HC correlated positively with total body mass (r values between 0.38 and 0.76, p<0.05), total lean body mass (r values between 0.37 and 0.74, p<0.05), as well as with the biopsied leg lean tissue (r between 0.42 and 0.75, p<0.05). Interestingly, only in MS patients, fiber type II and IIa CSA were negatively correlated with the fat percentage of the biopsied leg (r = −0.4 and −0.45, respectively, p<0.05).

Furthermore, muscle fiber type IIa and IIx CSA were negatively correlated with age in MS and HC (r values between −0.40 and −0.51, p<0.05). No correlations were detected between muscle fiber CSA and total fat mass and total fat percentage, in both groups. Finally, in case of MS patients, there was no correlation between muscle fiber CSA and EDSS or type of MS.

## Discussion

This study compared skeletal muscle characteristics of 34 MS patients and 18 matched HC and indicated quantitative as well as qualitative changes in the skeletal muscle characteristics of mildly affected MS patients. In particular, mean muscle fiber CSA, as well as CSA of type I, II and IIa fibers were significantly smaller in MS patients, independent of MS type and disease severity. Furthermore, MS patients showed reduced leg extensor muscle strength and tended to have a higher leg fat percentage and a lower leg lean tissue mass, compared to HC.

### Muscle fiber CSA

In accordance with the present work, Kent-Braun et al. and Garner et al. reported reduced muscle fiber size in 9 and 7 MS patients, respectively [5], [6]. Furthermore, in male MS patients we found a muscle fiber CSA hierarchy of type I and IIa>IIx, which differed from the hierarchy of CSA type IIa>I and IIx, seen in our healthy men. In female MS patients a muscle fiber CSA hierarchy of type I>IIa>IIx was found, which also differed from the patterns of CSA type I and IIa>IIx, seen in our healthy women. These differences suggest a selective type II(a) atrophy in MS patients, as also indicated by others [5], [6], [14]. Selective type II atrophy is considered to be an effect of ageing [25] or inactivity [26], [27]. Interestingly, in the present study average age was similar between MS patients and HC, suggesting that the reported observations could be a consequence of MS-induced physical inactivity, as already reported by others [28]–[30]. Contrary to the present study and the work of Kent-Braun et al. [5] and Garner et al. [6], another study did not report alterations in muscle fiber CSA in MS patients and HC [7]. These differences could be explained by the small sample sizes and/or the use of other techniques.

### Muscle fiber proportion

The present study also showed a tendency towards a higher proportion of type IIa fibers, at the expense of type I fibers, as previously reported in MS patients [5] and in immobilized healthy controls [15]–[17], suggesting that inactivity plays a role in MS. Other studies reporting muscle fiber proportions in MS and HC showed inconsistent results [6], [7]. These differences could be related to the use of different muscle tissue (vastus lateralis vs. tabialis anterior), the different anatomical functions of these muscles and the inclusion of patients with different levels of disability, making it difficult to draw solid conclusions.

### Body composition

Body composition, body fat and lean tissue mass in particular, has already been investigated in MS [31]. Similar to our results, some studies also reported similar total body fat percentages and total body lean tissue mass in MS patients and HC [10], [32]–[35] and a slightly higher fat percentage and lower lean tissue mass in the lower extremities of (female) MS patients [32]. However, the work of others [10], [32]–[35] was never correlated to muscle fiber CSA, making it difficult to further compare between studies. In addition, the present study showed that muscle fiber CSA was positively correlated with lean tissue mass and negatively correlated with fat percentage of the biopsied leg, indicating that the preservation of muscle mass of the lower limb of MS patients is very important, already at the early stage of the disease.

### Muscle strength

The reduced maximal isometric quadriceps strength of the MS patients was consistent with previous findings [6]–[9], [12], [13]. Furthermore, muscle fiber CSA was highly correlated with muscle strength of the quadriceps, suggesting that reduced CSA contributes to muscle weakness in MS patients and that changes in skeletal muscle characteristics in MS may affect function.

### Limitations

The muscle fiber CSA and proportion are considered to be representative for the whole muscle under investigation. However, it should be kept in mind that the fiber type proportion changes along the length and the depth of the muscle. Therefore, Lexell et al. [36], [37] recommended to collect three biopsies from different depths of the muscle and to analyse >150 fibers from each sample to reduce sampling error. Given the ethical concerns we collected only one biopsy and analysed approximately 170 fibers from each sample, being aware of the variation in our study results. Nevertheless, a post hoc power analysis showed that each group should comprise at least 19 subjects, to detect a difference of 20% between mean muscle fiber CSA of HC and MS patients (power  = 0.8, p = 0.05), indicating an appropriate sample size in the present study. Furthermore, given the cross sectional nature of the study, these results do not allow conclusions on causality.

## Conclusions

In conclusion, the results of this study suggest that muscle fiber characteristics are altered by MS, irrespective the type or severity of the disease. This emphasises the need for rehabilitation programs focusing on preservation and/or rebuilding of muscle mass of the lower limb, as a means to protect and/or enhance physical function in MS patients.
